# Methods Military and Medical

**Published:** 1916-05-13

**Authors:** 


### The Hospital

The Workers' Newspaper of Administrative Medicine and Institutional Life, Administration, National Insurance and Health.

No. 1561, Vol. LX. SATURDAY, MAY 13, 1916. PRICE ONE PENNY.

### METHODS MILITARY AND MEDICAL.

Within the last year or so large numbers of medical practitioners who had previously been engaged in civil practice have entered the Army, and have thus come under the discipline of military law and custom. The change for the individual is usually a considerable one, and he may well find that some of the demands of the new situation have certain features which prove irksome to him. There are, however, considerations which rule the position, and which, if constantly borne in mind, will go far to breed patience and equanimity. First and foremost is the fact that service has been taken, not for individual profit and advantage but for the larger interests of King and country. It is the day of sacrifice for a great national end, and the greater the sacrifice the greater the honour. In this order of events the medical profession stands in a very prominent place, for without efficient medical help armies in the field are impossible. Hence there arises this further thought, that every medical man who has given up his civil livelihood in order to serve the Army is a representative to the nation of the profession to which he belongs, and such a position is not without its proud and stimulating inspiration. By the readiness and good will with which he adapts himself to the demands of his new position he announces the anxiety and ability of the profession to serve effectively the country at the hour of a great national emergency. Once more, his new duties give him the opportunity to render direct assistance to those who bear the heat and burden of the day and who stand at the risk of their lives in their country's defence. Against the incidence of disease he can afford some measure of scientific protection, while for the sick and wounded he has all the resources of the healing art. When these various motives are combined it must be recognised that they form no small moral support, whether this is needed in hours of wearisome monotony or amidst the dangers of the stricken field. For both the one and the other the practitioner has volunteered under the claim of duty, and of the justice of his cause he can have no doubt. He carries his beneficent art where there is urgent need for it, and amidst wounded and helpless men no narrow bounds of nationality will stay his hand. That he is one with his countrymen in the cause which they know to be right is, of course, true, but in his special position he is in a particular measure conscious of the claims of humanity.

Prominent amongst the new influences of which the civil practitioner who has taken service with the Army becomes conscious is the sense of organisation and discipline. Hitherto his atmosphere has been one of freedom both of judgment and of action. Now he becomes part of a great machine, in which he has to play a part selected, not by his own choice or inclination but by the orders of others. Upon the wisdom or necessity of these orders he may think as he will, but he must needs obey them. Whereas in his former work he selected his authorities he has now to accept those which are imposed on him. And not infrequently he will feel that the authority arises less from professional knowledge and ability than from military rank and position. The change of atmosphere is an acute one, and there must needs arise from it a sense of strangeness and often a demand for much self-control. He has not ceased to be a medical practitioner, but at the same time he has become a soldier, and hence he must accommodate himself to military standards and must cultivate the military virtues, among which personal subordination and unquestioning obedience occupy a high place. There will still be some room for the independent judgment and personal decision which were so valuable in his former work, but often he must suppress these under the influence of authoritative orders. Taking the whole position into view, it is obvious that there are occasions not a few in which friction might well arise, but what has to be remembered is that friction is the one thing which cannot be tolerated in the machinery of an organisation the purpose of which is, not the exaltation of the individual but the success of a declared enterprise. There must be sacrifice of personal freedom in order that a greater end may be gained. Here in a word is the sharp lesson and contrast which is offered to the civil practitioner when he enters on military duties.

One experience which a number of the new military medical officers have found very trying is their appointment to duties which afford no opportunity for the application of the special knowledge and ability they happen to possess. They have perhaps spent some years in the cultivation of a particular branch of practice, operative or otherwise, and they find themselves condemned to work which seems commonplace or routine. Very naturally, and by no means for purely selfish reasons, they feel that their full and true value is not being utilised, and that the Army is not receiving the measure of service they are capable of rendering. Such a situation is not an easy one to sustain with good grace, yet time and tact may often do something to redeem it. But it has to be accepted, and prudence and patriotism both dictate that it should be accepted with a willing spirit. After all, the whole purpose of this medical volunteering is not to add to the experience of the individual practitioner nor to afford him opportunities for the display of any particular skill he may possess. He has taken service in the national cause, but the exact place and character of this service must be left to those who have to view the situation as a whole and to bear the responsibility of it. Lastly, there may be justly remembered the extraordinary and altogether unexpected demands which have been made on the organisation of the Medical Department of the War Office by the unprecedented development of our fighting Forces. Never until the outbreak of the war was it even imagined that this country would put into the field armies which would be numbered by millions. Yet, on universal testimony, it is agreed that the Medical Services have not failed to rise to the occasion, partly as a result of efficient organisation and control and partly as a result of individual sacrifices. Everything, it is said, comes to him who knows how to wait. The generalisation, doubtless, has its exceptions. But this at all events is certain, that none of the new medical officers, however obscure the sphere in which he is placed, need return to civil life without the consciousness that in the midst of great events he tried to do his duty.

